# Bidirectional meta-Kronecker factored optimizer and Hausdorff distance loss for few-shot medical image segmentation

**DOI:** 10.1038/s41598-023-35276-4

**Published:** 2023-05-19

**Authors:** Yeongjoon Kim, Donggoo Kang, Yeongheon Mok, Sunkyu Kwon, Joonki Paik

**Affiliations:** 1 Department of Artificial Intelligence, Chung-Ang University, 84 Heukseok-ro, Seoul, 06974 Korea; 2 Department of Image, Chung-Ang University, 84 Heukseok-ro, Seoul, 06974 Korea

**Keywords:** Diseases, Health care, Medical research, Mathematics and computing

## Abstract

To increase the accuracy of medical image analysis using supervised learning-based AI technology, a large amount of accurately labeled training data is required. However, the supervised learning approach may not be applicable to real-world medical imaging due to the lack of labeled data, the privacy of patients, and the cost of specialized knowledge. To handle these issues, we utilized Kronecker-factored decomposition, which enhances both computational efficiency and stability of the learning process. We combined this approach with a model-agnostic meta-learning framework for the parameter optimization. Based on this method, we present a bidirectional meta-Kronecker factored optimizer (BM-KFO) framework to quickly optimize semantic segmentation tasks using just a few magnetic resonance imaging (MRI) images as input. This model-agnostic approach can be implemented without altering network components and is capable of learning the learning process and meta-initial points while training on previously unseen data. We also incorporated a combination of average Hausdorff distance loss (AHD-loss) and cross-entropy loss into our objective function to specifically target the morphology of organs or lesions in medical images. Through evaluation of the proposed method on the abdominal MRI dataset, we obtained an average performance of 78.07% in setting 1 and 79.85% in setting 2. Our experiments demonstrate that BM-KFO with AHD-loss is suitable for general medical image segmentation applications and achieves superior performance compared to the baseline method in few-shot learning tasks. In order to replicate the proposed method, we have shared our code on GitHub. The corresponding URL can be found: https://github.com/YeongjoonKim/BMKFO.git.

## Introduction

Medical image segmentation plays a key role in numerous clinical applications, including diagnosis and treatment assistance, surgical planning, and disease progression monitoring. Precise delineation of organs and lesions from medical modalities, such as MRI and CT scans, is essential for these applications. For example, accurate tumor segmentation is crucial for gauging disease extent, devising radiation therapy strategies, and evaluating treatment efficacy in oncology. In addition, the segmentation of organs such as the brain, liver, and heart can aid in surgical planning and monitoring disease progression. Therefore, medical image segmentation is a critical component of modern healthcare, necessitating the development of precise and efficient algorithms to enhance patient outcomes.

Traditional image segmentation methods, such as region growing, graph cut^1^, active contour model^2^, and active shape model^3^, have been widely used in medical image analysis and have produced reliable segmentation results in many clinical applications. Software using these methodologies includes 3D Slicer, ITK-SNAP, and BrainSuite. However, these methods have limitations such as sensitivity to noise, computational cost, and the need for manual intervention.

Owing to recent advancements in deep learning technology, the field of semantic segmentation has seen substantial progress in various computer vision applications, including medical image segmentation. The U-Net^4^ model-based semantic segmentation method has emerged as a key tool for various clinical procedures and medical imaging research using MRI or CT. Therefore, it is necessary to develop a deep-learning-based application to complement the limitations of the traditional methodologies.

Recent advances in deep learning have become an inflection point for AI to achieve human-level performance. Before the deep learning era, the expert system^5^ is a representative computer-based system that complements human decision-making. However, with the arrival of the era of deep learning, research beyond hand-craft-based methods has been conducted in various fields. In particular, FCN^6^, U-Net^4^, DeepLab v3 plus^7^, and SegNet^8^ models have improved the performance of semantic segmentation. These works are widely applied to medical image segmentation required for image reading and diagnosis in the medical field^9–13^. The reason is that medical data requires medical experts, which incurs high costs for collection and annotation. Like other fields of research, it also raised a common problem that good results are obtained when learning with a lot of data. Therefore, massive data in medical image segmentation is a crucial part in research and often becomes an important factor that makes it difficult to achieve the purpose.

In the semantic segmentation method of medical images, problems with such a large amount of data are summarized as follows.

- In the field of medicine, a fundamental challenge arises from the scarcity of data required for research, as data from healthy subjects are readily available, while acquiring patient data proves to be difficult.
- The disclosure of patient’s medical data for research and the privacy policy of patients conflict with the ethical aspects of medical researchers, which causes another problem that makes research more difficult.
- Medical imaging data requires expertise in the classification and judgment of diseases, and it induces a lot of cost and difficulty in the annotation.

**Figure 1:**
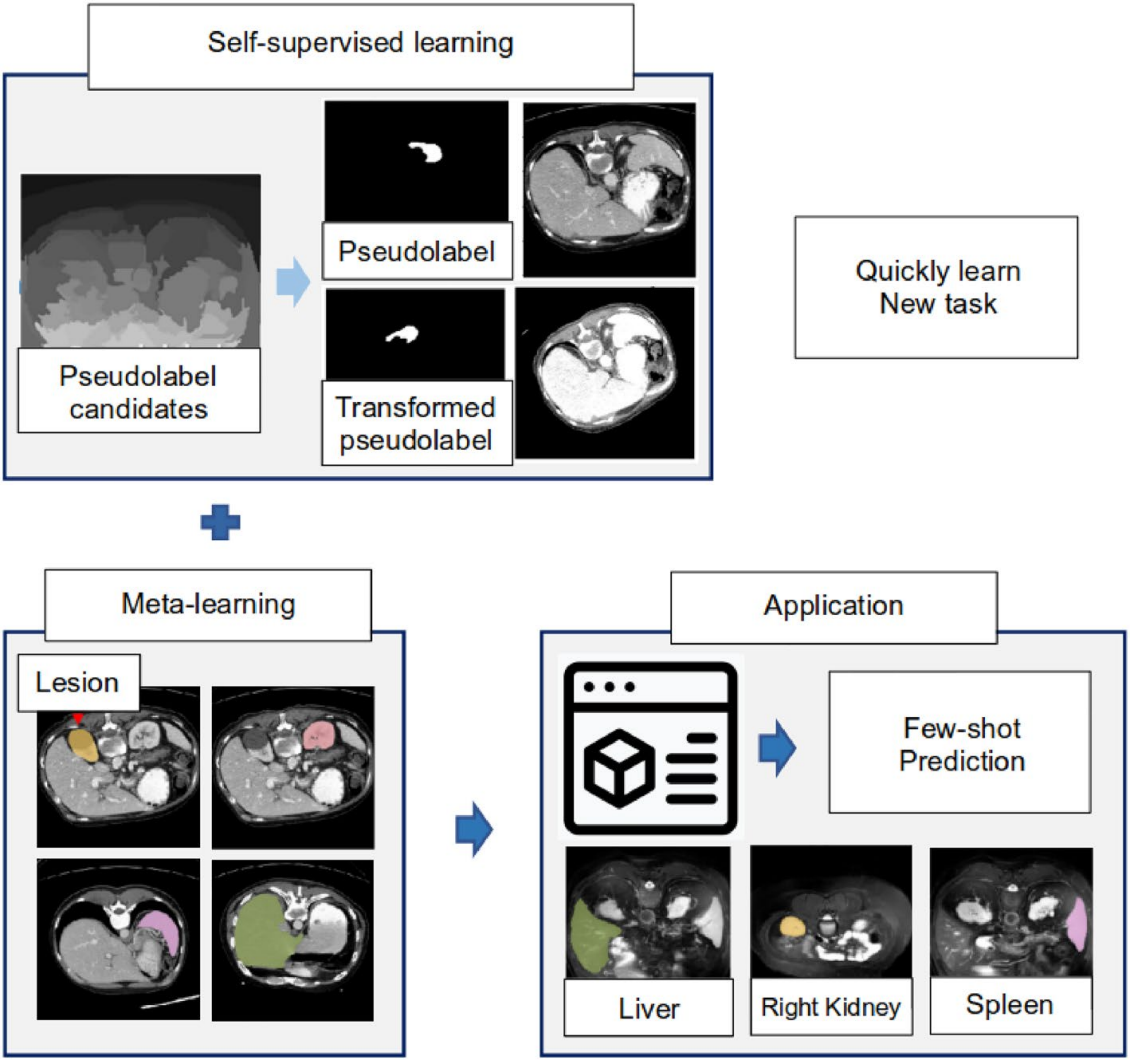
Overall architecture of the proposed model.

To solve these problems, few-shot learning^14–19^ methods that learn only use a limited dataset have been proposed. Few-shot learning predicts unlabeled query sets by quickly optimizing the model with only a small number of labeled support sets. Therefore, applying few-shot learning to medical images has the advantage of achieving efficient image segmentation with only a small amount of data in order to semantically segment invisible lesions. As shown in Fig. 1, superpixels perform self-supervised learning by creating pseudolabels and solve the problem of data shortage with meta-learning.

Recently, meta-learning methods are attracting attention to utilize these technologies^20–22^. Meta-learning is well known as “learning how to learn”. Because meta-learning teaches the learning process to solve a problem, it is important in that it aims to implement universal AI. Various meta-learning approaches are divided into model-based approaches^20^ widely used in reinforcement learning, metric-based approaches used for data clustering in embedding spaces^21^, and optimization-based approaches^22^ that optimize initial parameters. In this paper, we used the optimization-based approach of model-agnostic meta-learning (MAML) to find the generalized optimal initial point by considering model parameters and meta-optimizer simultaneously^22^. In addition, we used the Kronecker-factorized decomposition algorithm to efficiently decompose network parameters^23^. Therefore, the proposed method can optimize the (tens of thousands) parameters of the network stably and show better performance than MAML algorithm only^23^.

MAML only updates optimal initial values of model-parameters and meta-optimizer parameters. However, this approach does not make the best use of limited data. To solve this problem, we propose a novel meta-optimizer algorithm that considers validation and test set as well as train set in meta-learning. The proposed meta-optimizer consists of two optimizers which are updated separately: meta-train optimizer and meta-test optimizer. Through few-shot learning, the parameters of a few-shot learner are only updated with a *k*-shot support set. It leads to a highly unstable learning curve and consequently hard to optimize. To handle this difficulty, we use a meta learning scheme. The original MAML finds optimal initial values on the inner loop, which updates parameters of meta optimizer independently. Inspired by this scheme, we organize the inner loop to train-validation-test separately, then parameters of the meta optimizer are updated like few-shot learning procedure. We called the train phase of inner loop as a meta-train optimizer and the validation-test phase as meta-test optimizer. By this new inner loop design, we can train robust few-shot learner.

In the semantic segmentation of medical images, the conformation between Ground Truth (GT) and prediction is important. To handle this issue, we introduce the Hausdorff distance loss as our objective function. It measures the distance of the segment’s boundary and minimizes them. Therefore, this objective function focuses on the shape of the segment rather than simply the area of the intersection.

The contributions of this paper are summarized as follows:

- We proposed an optimization-based meta-learning algorithm to few-shot medical image segmentation and novel algorithms that optimize few-shot like meta-learner consisting of meta-train optimizer and meta-test optimizer.
- We used a Kronecker-factored decomposition algorithm to compute optimal points of important learning parameters in optimizer-based meta-learning. This outperforms the baseline in few-shot learning with efficient parameter computation with MAML.
- We used Hausdorff-distance loss that focus on the shape of segment rather than area. It reduces noise of prediction and shows favorable results, especially in qualitative aspects that are important to assist a doctor’s judgment.

The paper is organized as follows: After the “Introduction” section, we briefly summarize “Related work” section. The “Proposed method” section include in-depth description of model-agnostic meta-learning with Kronecker-factored decomposition and Hausdorff loss. After presenting “Experimental results” section with both quantitative and qualitative evaluation on kidney, liver, and spleen images, we concluded the paper.

## Related work

### Meta-learning

The goal of meta-learning is to train a model on different tasks so that it can solve unseen tasks using only a few training samples. Each task consists of dataset $D \in {\mathbb {R}}^{2}$, and optimal model parameters are computed by:

1 $$\begin{aligned} \theta = {\arg } \underset{\theta }{\min } {\mathbb {E}}_{D\sim p(D)}\left[ {L_{D\sim p(D)}^{\mathrm{validation}}}\left( \theta - \alpha \nabla {L_{D\sim p(D)}^{\mathrm{train}}}(\theta )\right) \right] , \end{aligned}$$

where $\theta$ represents the set of model parameters, ${\mathbb {E}}{D\sim p(D)}$ the expectation over a distribution of dataset *D*, and ${L}(\theta )$ the loss function for a given dataset *D* and model parameters $\theta$. $\theta - \alpha \nabla {L_{D\sim p(D)}^{\mathrm{train}}}(\theta )$ denotes the gradient update for training data, where $\alpha$ represents the learning rate, and $\nabla {L_{D\sim p(D)}^{\mathrm{train}}}(\theta )$ is the gradient of the loss function *L* with respect to $\theta$ on the training data. ${L_{D\sim p(D)}^{\mathrm{validation}}}(\theta - \alpha \nabla {L_{D\sim p(D)}^{\mathrm{train}}}(\theta ))$ represents the loss function on the validation data, where $\theta - \alpha \nabla {L_{D\sim p(D)}^{\mathrm{train}}}(\theta )$ is the updated parameter using the training data. We compute the loss function on the validation data using the updated parameter. Thus, Optimizing the minimized ${\arg \min }$ $\theta$ becomes the objective function. Although this formula looks similar to the general learning method, we need to find a model parameter that is optimized for all *n* tasks since each task is different here. This *learning-to-learn* meta-learning typically includes metric-based^21^, model-based^24–31^ and optimization-based^22^ approaches.

The goal of metric-based meta-learning is similar to nearest neighbors (*k*-NN) and kernel density estimation algorithm^32^. First, *k* support sets and query set are projected onto the embedding space through the feature extractor, and then learned in the direction to minimize the metric or distance between the support set and the query set.

On the other hand, the core of model-based meta-learning is to design a model architecture specific to fast learning. This approach aims to update its parameters rapidly with a few training steps. Shaban et al. proposed a fully convolutional network (FCN)-based few-shot segmentation method^24^, which produces a set of parameters using a pre-trained feature extractor learned by a support set and then multiply to parameters from FCN that passed query image. Kate et al. proposed a conditional network for few-shot segmentation^25^, which is conditioned on an annotated support set of images via feature fusion to inference on an unseen query image. Recently, attention-based approach^26,27^ and graph convolutional network-based approach^28,29^, have become popular as research subjects because of their efficient use of feature relationships. In addition, the method using the global correlation network with discriminative embedding (GCN-DE)^33^ and the method using the location-sensitive local prototype network^34^ show impressive results in the few-shot task. Sun et al. proposed a medical image segmentation method that incorporated discriminative embedding into a global correlation network. This method employs a deep convolutional network trained episodically, incorporating an efficient global correlation module to capture the correlation between support and query images. Furthermore, it enhances the discriminability of deep embedding for efficient feature domain clustering. Yu et al. proposed a location-sensitive local prototype network, a prototype-based few-shot segmentation method that leverages spatial priors in medical imaging, addressing the need for large amounts of expert-level annotated data. Their methodology involves two main steps: (1) location-sensitive local prototype extraction and (2) grid-based few-shot segmentation. Their approach divides the challenging problem of global image segmentation into smaller, more manageable sub-problems of local region segmentation. The proposed method is different in that it focuses on the meta-learning algorithm and the objective function. Wang et al. proposed a network that uses both metric-based and model-based meta-learning methods jointly called PANet^30^, which learns class-specific prototype representations from support sets into an embedding space and then performs segmentation over the query set by matching each pixel to the learned prototypes. Ouyang et al. proposed a method using self-supervised learning exploits to superpixels called SSL-ALPNet^31^, which is especially effective in medical imaging because most CT or MRI images are suitable to apply a superpixel algorithm.

Optimization-based meta-learning methods assume that there are some internal representations that are more transferable than others. Under this assumption, it might learn internal features that are widely acceptable to all tasks rather than a single task. The pioneering work in this aspect is MAML^22^, which aims to find model parameters called meta-gradient that are sensitive to changes in the task, such that small changes in the parameters will produce large impacts on the loss function of any task. The meta-gradient update involves a gradient through a gradient. More detailed explanation will be given later.

### Kronecker decomposition

There are many different matrix decomposition or factorization schemes related to solving systems of linear equations, such as Cholesky factorization, QR decomposition, and LU decomposition. The purpose of decomposition is to implement efficient matrix algorithms. For example, when solving linear equations $Ax = b$, the matrix *A* can be decomposed using LU decomposition, which factorizes a matrix into a lower triangular matrix *L* and an upper triangular matrix *U*. The systems $L(Ux) = b$ and $Ux = L^{-1} b$ require fewer additions and multiplications to solve than original equations.

Denton et al. proposed applying truncated singular value decomposition to the convolutional neural network^35^, which is one of the earliest works to apply decomposition scheme to a neural network. Kronecker decomposition is another factorization scheme. It can replace a large matrix with two smaller Kronecker factor matrices that best approximate the original matrix. This decomposition scheme effectively reduces model parameters and consequently dimensionality. These properties of the matrix decomposition scheme are similar to the goal of meta-learning. Decomposition is interpreted as noise reduction and extract principle components. Therefore, matrix decomposition schemes assist to find the optimal meta initial point in a quick and stable manner.

### Hausdorff distance

Loss functions play an important role in determining the model performance. There are various loss functions based on the distance to measure the quality of model prediction. Although many deep learning models adopt cross-entropy loss function, the cross-entropy loss is not suitable for segmentation task in that it only considers intersection area, not the whole object shape. Therefore, in some cases, quantitative evaluation is high, but qualitative evaluation often shows poor results. To solve this problem, Sudre et al. proposed dice loss^36^, which is inspired from dice coefficient, a metric to evaluate segmentation results. It considers the region and scale of the predicted segment. To consider the segment’s boundary, Hayder et al. devise shape-aware loss^37^, which is a variant of cross-entropy loss by adding a shape based coefficient used in case of segment boundary. The average Hausdorff distance is widely used for a measure of two points sets^38^. Inspired by this concept, Hausdorff distance loss was designed to compare ground truth segmentation boundary and predicted segmentation boundary^38^. The directed Hausdorff distance from boundary point set *X* to *Y* is given by the sum of all minimum distances from all points from *X* to *Y*. Average Hausdorff distance can be computed by the bidirectional form of directed Hausdorff distance $D(\cdot ,\cdot )$, such that mean of *D*(*X*, *Y*) and *D*(*Y*, *X*).

## Proposed method

The proposed model-agnostic method is compatible with any other models without network component manipulation. We employed a state-of-the-art super-pixel-based self-supervised learning method, SSL-ALPNet^31^, as our baseline network for few-shot abdominal organ segmentation in both CT and MRI. In order to highlight the significance of meta-learning in medical imaging segmentation, we provide a concise overview of the distinctive features of medical imaging. CT and MRI images are acquired using highly sensitive medical equipment. Although each acquisition process is slightly different, it usually takes 10–15 min to obtain the result. Since each process has a unique characteristics, such as breathing patterns, movement, and slight body tremors, the correspondingly acquired image is also unique. Unfortunately, the uniqueness makes image segmentation difficult, and degrades the optimization performance of few-shot learning. To solve this problem, we proposed a bidirectional meta-Kronecker factored optimizer (BM-KFO) framework.

**Figure 2:**
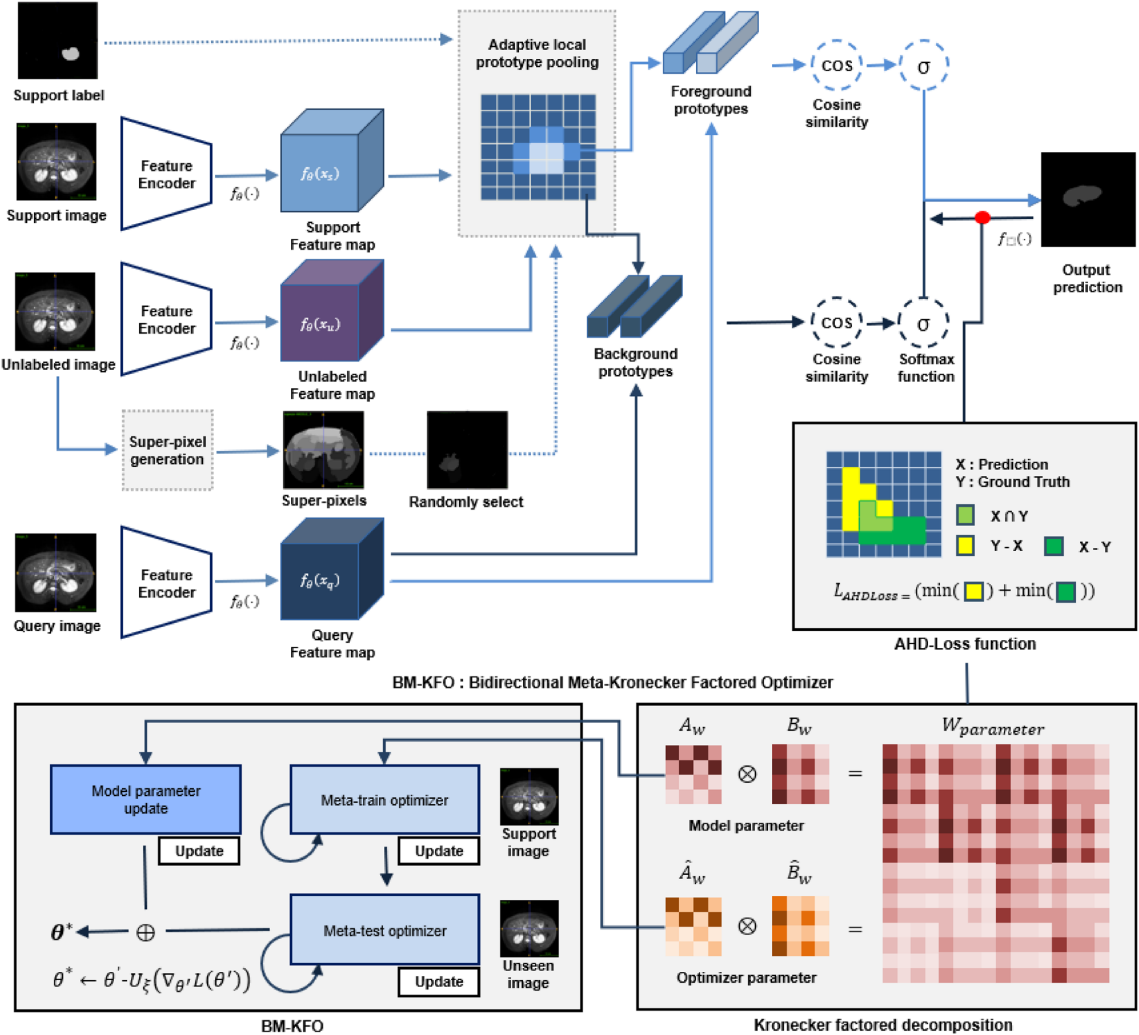
Overall architecture of the proposed model.

Figure 2 the overall framework of the proposed model, which uses ResNet as a feature extractor, and takes an MRI image as input. Although a general few-shot learning model uses only support images to train the network, we additionally use self-supervised learning scheme with super-pixel pseudo labels to use a limited number of data. Each pseudo label is created from the super-pixel version of support images. The model is then trained using both original support and pseudo labels. Each unlabeled image is also created as a super-pixel pseudo-label image. Then, the original image and pseudo-label are used as support and query sets^31^. Proposed BM-KFO framework falls into the category of optimization-based meta-learning for faster optimization of a few-shot task. The proposed framework is divided into meta-train and meta-test optimizers, and the parameters are efficiently and stably learned using kronecker-factored decomposition. We use objective function which combines cross-entropy and Hausdorff distance (HD) losses to minimize the difference between GT and prediction in the segmentation process. In the following section, we will describes the BM-KFO and HD loss, respectively.

### BM-KFO: bidirectional meta-Kronecker factored optimizer

#### MAML: model-agnostic meta-learning

The MAML algorithm is the basis of our method. We aim to find an objective function that optimizes the meta initial parameter $\theta$ which can be quickly applied to an unknown task. The training data, denoted as *D*, used for meta-learning include a support and query sets. The loss function with the meta-initial parameter $\theta$ is expressed as $L(\theta )$, and task-specific parameters are then computed as follows:

2 $$\begin{aligned} \theta ^{\prime } = {\mathbb {E}}_{D\sim p(D)}[(\theta - \alpha \nabla {L}_{D}(f_{\theta }))], \end{aligned}$$

where $\alpha$ represents the learning rate, and *p*(*D*) the distribution of each task’s training data. Meta-initial parameter is updated using loss which calculated by task-specific adapted parameters with gradient descent as:

3 $$\begin{aligned} \theta \leftarrow \theta - \beta \nabla _{\theta }\sum _{D \sim p(D)}L_{D_{i}}\left( f_{\theta _{\prime ^{i}}}\right) , \end{aligned}$$

where $\beta$ represents the meta step size. *p*(*D*) is the distribution of the data and the sampled new task $D_{i}$ is used to update the parameter $\theta$ of $f_{\theta }$. Thus, it can be explained in three steps: (1) Calculate the gradient of the loss function $L_{D_{i}}(f_{\theta _{\prime ^{i}}})$ concerning the model parameters $\theta '$, evaluated on the task data $D_{i}$, (2) compute the sum of the loss over all tasks sampled from *p*(*D*), and (3) determine the learning rate $\beta$ and update $\theta$. The basic algorithm of the same method as in the general case is summarized in Algorithm 1.
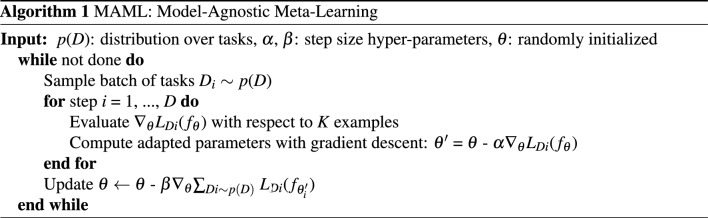


The inner loop statement optimizes the task-specific parameter $\theta ^{\prime }$ through gradient descent during meta-training. The outer loop parameter $\theta ^{\prime }$ is computed by the inner loop code. The meta-initial parameter is updated by gradient of loss function computed over each task, which is referred as *meta-testing*. By this inner-outer loop design, network can start optimization from generalized initial point. It is an algorithm for mata-testing with the optimal point $\theta ^{\prime }$ as input. $D_{i}$ is tested with unseen data, and better generalization is possible.

#### Meta-Kronecker factored optimizer

Inspired by a MAML algorithm called META-KFO^23^, we devised a meta-optimizer that is independent of the model parameters. The function $U_{\xi }$ is a meta-optimizer that defines for updating model parameters^23^. The meta-optimizer is to train the model parameters and optimizer parameters jointly. With optimizer parameters $\xi$, we use this optimizer to gradient updates:

4 $$\begin{aligned}{} & {} L(\theta ) = {\mathbb {E}}_{D\sim p(D)}\left[ loss_{D} \left( \theta - \alpha {U_{\xi }}\left( \nabla {loss_{D}}(\theta )\right) \right) \right] , \end{aligned}$$

5 $$\begin{aligned}{} & {} U_{\xi }(\nabla {loss(\theta )}) = f(\nabla {loss(\theta ){;}\;\phi }), \end{aligned}$$

where $L(\theta )$ represents the objective function, which is defined as the expectation over a distribution of datasets *D* drawn from a probability distribution *p*(*D*). The function $loss_{D}(\theta )$ represents the loss function for a particular dataset *D* and model parameters $\theta$. The term $\alpha {U_{\xi }}(\nabla {loss_{D}}(\theta ))$ denotes the update rule for the meta-parameters $\theta$, where $\alpha$ is the learning rate and $U_{\xi }(\nabla {loss_{D}}(\theta ))$ represents the update for $\theta$ obtained from the meta-optimizer with optimizer parameter $\xi$. In Eq. (5), $f(\cdot )$ represents a small parameterized neural network that does not increase the entire model parameters that are independent of the model parameter $\theta$. Where $\xi$ denotes the hyper-parameters of the function $U_{\xi }$ and $\phi$ are values used to optimize other hyper-parameters. Based on these two methods, we differentiated the parameters of the model and optimizer and constructed a meta-train optimizer and a meta-test optimizer. Unlike the MAML method, which only updates model parameters during learning, model parameters, and optimizer parameters are separated, and opti-parameters are simultaneously updated starting with the initial model parameters. These updates are applied to the meta-train optimizer and the meta-test optimizer, respectively, and finally, the updated parameter creates $\theta ^{*}$. The proposed method can be found in the Bidirectional meta-Kronecker factored optimizer (BM-KFO) section.

META-KFO provides better meta-learning capabilities with a shallow network. However, META-KFO is an external meta-optimizer that works inside the network. Therefore, the meta-optimizer $U_{\xi }$ have tens of thousands of parameters, which directly impacts to the network complexity. To reduce the computational complexity and memory usage, we used the Kronecker product to decompose the matrix as a convenient representations. Specifically, given any $m \times n$ matrix *A* and $p \times q$ matrix *B*, the Kronecker product between *A* and *B* is defined as:

6 $$\begin{aligned}{} & {} A = \left[ \begin{array}{ccc} A_{11} &{}\quad \dots &{}\quad A_{1n} \\ \vdots &{}\quad \ddots &{}\quad \vdots \\ A_{m1} &{}\quad \dots &{}\quad A_{mn} \\ \end{array} \right] \in Mat(m,n;{\mathbb {R}}), \quad B = \left[ \begin{array}{ccc} B_{11} &{}\quad \dots &{}\quad A_{1q} \\ \vdots &{}\quad \ddots &{}\quad \vdots \\ A_{p1} &{}\quad \dots &{}\quad A_{pq} \\ \end{array} \right] \in Mat(p,q;{\mathbb {R}}), \end{aligned}$$

7 $$\begin{aligned}{} & {} A \otimes B \in Mat(mp, nq; {\mathbb {R}}), \quad A \otimes B = \left[ \begin{array}{ccc} A_{11}B &{}\quad \dots &{}\quad A_{1n}B \\ \vdots &{}\quad \ddots &{}\quad \vdots \\ A_{m1}B &{}\quad \dots &{}\quad A_{mn}B \\ \end{array} \right] \in {\mathbb {R}}^{mp \times nq}, \end{aligned}$$

where *A* is an $m \times n$ matrix, and *B* is a $p \times q$ matrix. $A \otimes B$ denotes the Kronecker product of *A* and *B*, which is a matrix of size $mp \times nq$ with real entries. The entries of $A \otimes B$ are obtained by taking each entry $A_{ij}$ of *A* and multiplying it by the entire matrix *B*. More specifically, the (*i*, *k*)-th entry of $A \otimes B$ is given by $(A \otimes B)_{ik} = A_{ij}B_{kl}$, where *i* and *j* are such that $1 \le i \le m$ and $1 \le j \le n$, and *k* and *l* are such that $1 \le k \le p$ and $1 \le l \le q$.

The parameters of the optimizer are decomposed to the following Kronecker factorization.

8 $$\begin{aligned} W = R^{T} \otimes L \in {\mathbb {R}}^{m \times n}, \; {\mathrm{for}}\; R \in {\mathbb {R}}^{m \times n} \;{\mathrm{and}}\; L \in {\mathbb {R}}^{n \times n}. \end{aligned}$$

    Among several different decomposition methods, we used Kronecker-factored decomposition since: (1) the computational and memory cost of the Kronecker-product are acceptable, (2) $R^{\intercal } \otimes L$ is full-rank whenever *L*, *R* are full-rank, and (3) the identity matrix lies in the span of Kronecker-factored matrices. In particular, this last motivation allows meta-optimizers to recover the gradient descent update by letting *R*, *L* be the identity^23^. More specifically, in Kronecker-factored decomposition, the rank is closely associated with the dimensionality reduction of the decomposed tensor. When the learning parameters are decomposed and the rank of the decomposed tensor is lower, dimensionality reduction and information compression takes place. In other words, it is possible to reduce unnecessary information and obtain core information. On the other hand, if the rank of the decomposed tensor is full-rank, the dimension is equal to the size of the original tensor, and the original tensor can be completely recovered. Therefore, rank plays a major role in terms of information loss and efficient decomposition in Kronecker-factored decomposition. Lower ranks do not perfectly reproduce the original dimensionality, so a balance must be struck between dimensionality reduction and loss of information.

The effect of the number of convolutional layers on the adaptation performance can be compared with the KFO and the MAML method. First, the performance of both methods improves as the model size increases. In addition to better meta-learning, the improvement may come from an increase in the capacity of the model to learn the target task. Second, the net gain of meta-kfo tends to decrease with increasing layer size. In other words, the advantage of directly transforming the gradients using an external meta-optimizer is reduced because the upper layers of the larger model have more capacity to meta-learn to control their lower layers. Third, it is more performance to use Kronecker decomposition of parameters compared to MAML^23^.

#### Bidirectional meta-Kronecker factored optimizer (BM-KFO)

In the inner loop of MAML, Meta-KFO and other meta-learning frameworks only computes meta-optimizer parameters by the training loop. After obtaining the computed meta-optimizer parameters, a test loop in the inner loop assesses their effectiveness. To achieve this, we need to divide the *k* support sets into training and testing sets. While this method is appropriate for numerous *k*-shot situations, in low-shot learning contexts, such as the one-shot learning setting of our target dataset, we have only one training and testing set available for training the meta-optimizer. Consequently, in a low-shot setting, it is necessary to employ a test set within the inner loop. To solve this problem, we present a meta-learning framework consisting of meta-train optimizer and meta-test optimizer. It is inspired by a general learning process that train-validation-test and actor-critic algorithm in reinforcement learning^39^.
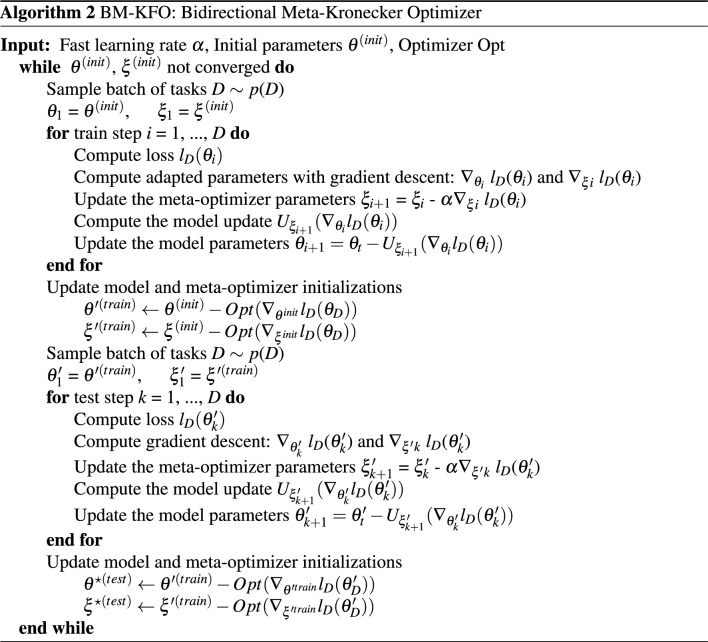


**Figure 3:**
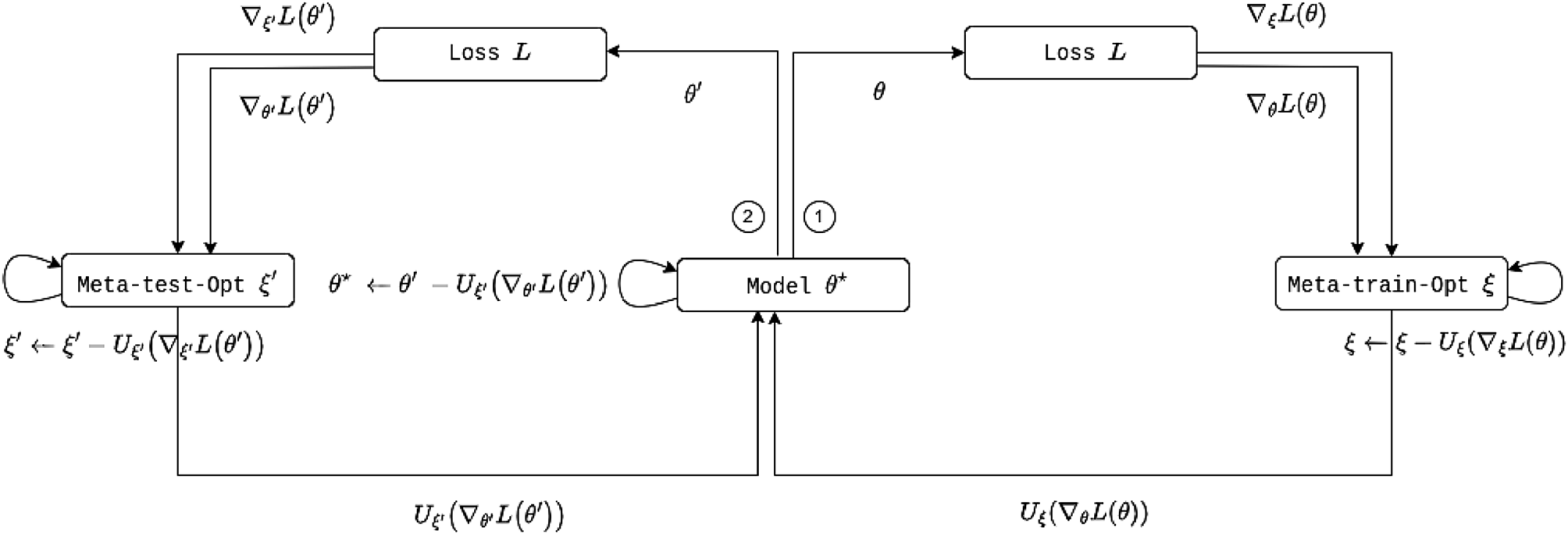
Bidirectional meta-Kronecker factored optimizer.

Algorithm 2 takes the parameters of the model and optimizer as input. Meta-train-optimizer and meta-test-optimizer are processed during batch. It is divided into two blocks $\theta ^{\prime }$ and $\xi ^{\prime }$, output parameters from meta-train feed to the next block as input. *For-loop* statements proceed in the form of updating meta-optimizer and model parameters, respectively. The output of two blocks, $\theta ^{\star }$ and $\xi ^{\star }$, respectively, are final meta-initial point. Figure 3 is visualization of Algorithm 2.

The proposed BM-KFO takes the form:

9 $$\begin{aligned} \theta ^{\prime } \leftarrow \theta - \beta \frac{\partial {L}(\theta )}{\partial \theta },\; \xi ^{\prime } \leftarrow \xi - \beta \frac{\partial {L}(\theta )}{\partial \xi }, \;{\mathrm{and}}\; {\mathrm{(BM-KFO): }} \theta ^{\star } \leftarrow \theta ^{\prime } - U_{\xi ^{\prime }}\left( \nabla _{\theta ^{\prime }}{Loss(\theta ^{\prime })}\right) , \end{aligned}$$

where $\theta ^{\prime }$ and $\xi ^{\prime }$ take $\theta$ and $\xi$ of each parameter as inputs, and produce $\nabla _{\theta }$ $Loss(\theta )$. This process is a meta-train-optimizer, and the process of entering $\theta ^{\prime }$ and $\xi ^{\prime }$ once again and finding $\theta ^{\star }$ becomes the meta-test-optimizer. $\theta ^{\star }$ is the final model parameter passed through meta-train optimizer and meta-test optimizer. The detailed BM-KFO framework pseudo-code is described in Algorithm 2, and the overall meta-optimizer flow is shown in Fig. 3.

### HD-loss: Hausdorff distance loss

Hausdorff distance is a representation of the difference in the boundary between GT and prediction as a distance. It measures the distance between the set of coordinates of the difference between GT and prediction, and is widely used in medical image segmentation. Average Hausdorff distance (AHD-Loss) is applied to measure the performance of various applications, including cerebrovascular segmentation^40^, extra-cranial facial nerve segmentation^41^. We used the average Hausdorff distance as our objective function to alleviate the unstable learning problem in few-shot learning. HD-Loss defines the mismatch of two sets as a nonlinear function that measures the distance. The max–min method using a general distance function is as follows:

10 $$\begin{aligned} D_{distance}(x, y) = || x - y ||. \end{aligned}$$

Given two sets $X = \{x_{1}, x_{2},\ldots ,x_{m}\}$ and $Y = \{y_{1}, y_{2},\ldots ,y_{n}\}$, the Hausdorff distance, denoted as *D*(*x*, *Y*), from a point in *X* to *Y* is defined as:

11 $$\begin{aligned} D_{distance}(x, Y) = \underset{y\in Y}{\min }\;d(x,y). \end{aligned}$$

The hausdorff distance from set *X* to *Y* is:

12 $$\begin{aligned} HD(X,Y) = \underset{x\in X}{\max }\;D_{distance}(x, Y) = \underset{x\in X}{\max }\;\underset{y\in Y}{\min }\;||x - y||. \end{aligned}$$

Conversely, the distance from set *Y* to *X* is:

13 $$\begin{aligned} HD(Y,X) = \underset{y\in Y}{\max }\;D_{distance}(y, X) = \underset{y\in Y}{\max }\;\underset{x\in X}{\min }\;||y - x||. \end{aligned}$$

When $Prediction = X, GT = Y,$ the final expression is as follows:

14 $$\begin{aligned} L_{HDLoss}(Pred, GT) = max(HD(Pred, GT), HD(GT, Pred)). \end{aligned}$$

#### AHD-loss: average Hausdorff distance loss

**Figure 4:**
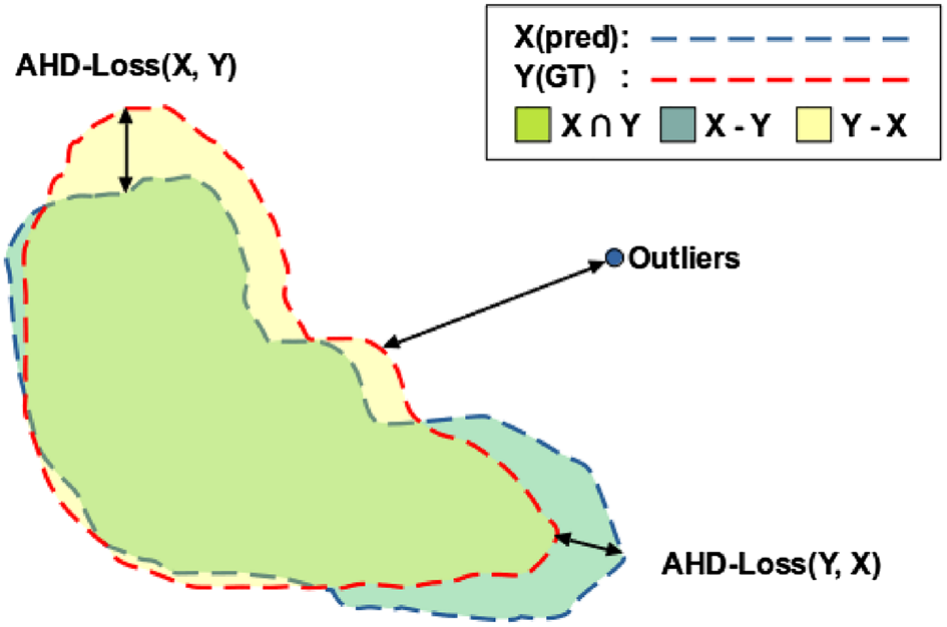
Visualization of AHD-Loss. It computes the distance between GT and prediction from both perspectives. Thus, it is robust to outliers.

The average Hausdorff distance between two finite sets of points *X* and *Y* is average of (14). HD-loss has a problem that it is sensitive to outliers. On the other hand, the average Hausdorff distance in the direction from point set *X* to *Y* is specified as the sum of all minimum distances from all points from point set *X* to *Y* divided by the number of points in *X*. The AHD-Loss can be calculated as both directions. Figure 4 is a visualization of AHD-Loss. It computes the distance between GT and predictions. It is form-aligned and robust to outliers than general Hausdorff distance. It is defined as follows using the AHD from *X* to *Y* and the AHD from *Y* to *X*:

15 $$\begin{aligned}{} & {} L_{AHDLoss}(X, Y) = \left( \frac{1}{X}\underset{x \in X}{\sum } \underset{y \in Y}{\min }\; D_{dist}(x,y)\;+\;\frac{1}{Y}\underset{y \in Y}{\sum }\ \underset{x \in X}{\min }\; D_{dist}(x,y)\right) /2. \end{aligned}$$

16 $$\begin{aligned}{} & {} D_{manhattan}(X, Y) = \sum _{i}^{n}(| x_{i},y_{i} |). \end{aligned}$$

17 $$\begin{aligned}{} & {} D_{euclidean}(X, Y) = \left( \sum _{i}^{n}\left( | x_{i},y_{i} |^{2}\right) \right) ^{1 \over 2}. \end{aligned}$$

18 $$\begin{aligned}{} & {} D_{chebyshev}(X, Y) = max_{i}(| x_{i},y_{i} |). \end{aligned}$$

As shown in (15), AHD-Loss calculates the difference between GT and Prediction from both perspectives, and can make the model focused more on the whole shape of the organ than intersections. It also robust to outlier points, and the distance measurement methods include Euclidean, Chebyshev, and Manhattan distances. Equation (16) is the Manhattan distance. It is also called *L*1 distance and it is equal to the sum of the differences of each coordinate. Equation (17) is the Euclidean distance. It is also called L2 distance, and it is the shortest distance between coordinates. Compared to the Eqs. (16,17) distances, we use Chebyshev distance because it can be measured the distance in all directions.

Our final objective function is sum of AHD- and cross-entropy losses.

19 $$\begin{aligned} L_{CE} = -\sum ^{n}_{i=1} y_{i}\;log(\hat{y}_{i}), \end{aligned}$$

where $y_{i}$ is GT and $\hat{y}$ is prediction.

20 $$\begin{aligned} L_{Total} = L_{CE} + L_{AHDLoss}. \end{aligned}$$

## Experimental results

### Implementation details

#### Dataset

To evaluate the proposed BM-KFO and Hausdorff distance loss, we used the abdominal MRI (Abd-MRI) segmentation dataset for training. It is part of Combined Healthy Abdominal Organ Segmentation Challenges^12^ from ISBI 2019^42^. CHAOS dataset can be downloaded via the following link. Go to the next URL: https://chaos.grand-challenge.org/Download/. It contains 40 different patients’ images of resolution $224\times 224$. This dataset does not include any tumors or lesions at the borders of the annotated organs of interest (i.e. liver, kidney, and spleen). The number of slices per person is between 26 and 50 (average 36). It provides 1594 slices for training and 1537 slices for testing.

#### Training

The proposed method was implemented with PyTorch based on SSL-ALPNet^31^. As a backbone for feature extraction, we used fully-convolutional ResNet101, which was pre-trained using part of MS-COCO dataset. Although ResNet101 is not the latest model, we did not have any modifications on it for a fair comparison with existing methods.

We used an NVIDIA RTX 3090 GPU for training network. The learning rate was 0.001 with a stepping decay rate of 0.98 per 1000 iterations with Stochastic Gradient Descent (SGD) and Adam optimizers. After 100,000 iterations, the proposed objective function converged, and it took about hours.

#### Evaluation metric

To evaluate the proposed method elaborately, we used *precision*, *recall*, and *dice coefficients* as metrics. Precision and recall are trade-offs. If the network predicts a very large prediction about 2–3 times than the ground truth, it is sufficient to cover all ground truth. In this case, precision becomes close to 1, meaning a near perfect match. On the other hand, recall is very low because its denominator is a number of predictions.

Precision, also known as positive predictive value and specificity, describes the purity of positive predictions relative to the ground truth. It measures how many predictions actually have matching ground truth segments.

21 $$\begin{aligned} {\mathrm{Precision}} = \frac{TP}{TP+FP} = \frac{TP}{\#\,{\mathrm{ground\,truth}}}. \end{aligned}$$

Recall, also known as sensitivity, effectively describes the completeness of our predictions relative to the ground truth. It measures all predictions given and the number that captures true positives.

22 $$\begin{aligned} {\mathrm{Recall}} = \frac{TP}{TP+FN} = \frac{TP}{\#\,{\mathrm{prediction}}}. \end{aligned}$$

Dice similarity, also known as F1 score or Sørensen–Dice index, is used to gauge the similarity of two segments. It is defined as the harmonic mean between precision and recall.

23 $$\begin{aligned} {\mathrm{Dice}} = \frac{2|X\cap Y|}{|X|+|Y|} = \frac{2TP}{(TP+FP)+(TP+FN)}, \end{aligned}$$

where |*X*| and |*Y*| represent the cardinality of segments.

### Quantitative evaluation

To evaluate 2D segmentation on 3D images, we follow the evaluation method established by^43^, where support and query sets are data of different patients. To evaluate the generalization ability in few-shot learning, the medical image segmentation method follows the evaluation method established by Roy et al. ’s setting 1^43^.

However, setting 1 has a problem in that the test class appears in the background of the training data during training. In other words, few-shot model learns each class respectively using inner-loop of BM-KFO. By updating meta-initial points computed by inner-loop, we can optimize quickly to other class. Each class is independently used for training, but input images are the same. Thus, the same input images cause unwanted spatial bias to the meta-optimizer. To handle this issue, we performed additional experiments with our method using setting 2, which removes test class relevant data. In setting 2, the model trained with the images in the test class is completely removed from the training data. In setting 1, label partitioning is unnecessary to train. On the other hand, in setting 2, data are divided into the upper and lower abdomen with liver, spleen, and left and right kidneys. It can make a separate group for testing. In each experiment, all the training data belonging to the test data group are removed. To simulate the sparsity of labeled data in applications, all experiments are conducted in one-way one-shot settings^31^.

Tables 1 and 2 show experimental results with setting 1 and setting 2, respectively. In both settings, the proposed method outperforms the other methods by a large margin. Especially, setting 1 provides relatively higher improvements than setting 2. Here, setting 1 and setting 2 refer to the setup to evaluate the generalization ability for unseen testing classes during training. Setting 1 corresponds to a scenario where the testing class can appear as the background of training data in a few-shot segmentation experiment for medical images. On the other hand, setting 2 refers to a situation in which the test class is completely invisible by excluding images that contain the testing class from the training dataset^31^. Table 2 presents two methods for few-shot medical image segmentation: SSL-RPNet^46^ and CRAPNet^45^. SSL-RPNet is a supervised approach that uses two core modules to capture local relational features and iteratively refines a segmentation mask. This method employs a prototype network with a context relational encoder, leading to improved performance in few-shot medical image segmentation. On the other hand, CRAPNet addresses the issue of neglecting the connection between the query set and support set, which is present in existing medical image segmentation studies using Prototype Networks. It represents the latest few-shot methodology that preserves pixel-related relationships between medical images.

**Table 1:** Experiment results(in Dice score) on abdominal MRI images under setting 2.

Method	Lower		Upper		Mean
	LK	RK	Spleen	Liver	
SE-Net^44^	62.11	61.32	51.80	27.43	50.66
Vanilla PANet^30^	53.45	38.64	50.90	42.26	46.33
ALPNet-init	19.28	14.93	23.76	37.73	23.93
ALPNet	53.21	58.99	52.18	37.32	50.43
SSL-PANet	47.71	47.95	58.73	64.99	54.85
SSL-ALPNet	73.63	78.39	67.02	73.05	73.02
CRAPNet^45^	74.66	82.77	70.82	**73.82**	75.52
Proposed method	**79.89**	**86.86**	**72.13**	73.43	**78.07**

Significant values are in [bold].

**Table 2:** Experiment results(in Dice score) on abdominal MRI images under setting 1.

Method	Kidneys				Mean
	LK	RK	Spleen	Liver	
SE-Net^44^	45.78	47.96	47.30	29.02	42.51
Vanilla PANet^30^	30.99	32.19	40.58	50.40	38.53
ALPNet	44.73	48.42	49.61	62.35	51.28
SE-FSS^43^	62.56	65.81	48.93	40.32	54.38
SSL-PANet	58.83	60.81	61.32	71.73	63.17
SSL-ALPNet	81.92	85.18	72.18	76.10	78.84
SSL-RPNet^46^	71.46	81.96	73.55	75.99	75.74
CRAPNet^45^	81.95	86.42	**74.32**	76.46	79.79
Proposed method	**84.14**	**87.07**	70.79	**77.42**	**79.85**

Significant values are in [bold].

The proposed method is compared with various state-of-the-art methodologies, including CRAPNet and SSL-RPNet. In setting 1, CRAPNet achieves a 4.3% higher performance than the proposed method for spleen segmentation, but the overall average is slightly lower. SSL-RPNet has lower overall performance than CRAPNet. In setting 2, CRAPNet shows an approximately 0.4% improved performance over the proposed method for liver segmentation.

### Ablation study

We carried out an ablation study that focused on three different perspectives, such as training time, loss function, and meta-learning method, to conduct a more comprehensive analysis of the proposed method.

Furthermore, we conducted additional experiments on training time per iteration, and training time to achieve convergence as shown in Table 3. We devised methods that integrate different combinations of components to ensure a fair comparison of each element of the proposed approach. Compared to the baseline method, the proposed method exhibits increased resource consumption in terms of memory and training time. This can be attributed to the extra inner loop that trains the meta-test optimizer and the computation time necessary for AHD loss. In contrast, MAML solely trains the meta-train optimizer in the inner loop.

**Table 3:** Evaluations on the various meta-learning methods and loss functions to the baseline and proposed method.

Method	Training time (s)	Method—baseline	Training time (ms/1iter)
SSL-ALPNet + CE	809	–	40.01
SSL-ALPNet + CE + Boundary loss	1114	305	55.71
SSL-ALPNet + CE + Boundary loss + HDLoss	1416	607	70.81
SSL-ALPNet + MAML	899	90	44.95
Proposed method	**1522**	**713**	**76.10**

The required memory usage during training (min–max), the required training time, and the time required for 1 iteration are compared. It is based on 20 epochs.

Significant values are in [bold].

Table 4 shows the result of experiments conducted to evaluate the impact of the loss function on both the baseline model and the proposed method. We utilized Recall, Precision, and Dice Score metrics to evaluate the performance of the proposed method. In each experiment, the baseline loss, cross-entropy loss (CE-Loss), boundary loss, and HD loss functions are compared. Boundary loss led to low Dice and recall scores but high precision scores due to its focus on the ground truth boundary. However, this led to the network struggling to fit irregular shapes, resulting in low recall scores. In the medical image segmentation task, identifying abnormal organ shapes is more crucial than normal ones. Combining Boundary loss with HD-Loss resulted in a slight increase in Dice and recall scores. Although the proposed method exhibits lower precision than other methods, it demonstrates high performance in terms of Dice, recall, and mean. This indicates that the proposed method produces more balanced outputs compared to other methods.

**Table 4:** Impact of each loss function on the baseline model and the proposed method, excluding the meta-learning approach.

Method	Dice score	Precision	Recall	Mean
SSL-ALPNet + CE	71.8	70.1	74.3	72.06
SSL-ALPNet + CE + Boundary loss^47^	66.3	90.3	52.9	69.83
SSL-ALPNet + CE + Boundary loss + HDLoss	69.4	**92.4**	56.2	72.66
Proposed method (+ CE + AHD-Loss)	**74.0**	70.6	**79.2**	**74.6**

Significant values are in [bold].

Table 5 presents a comparison of meta-learning methods including MAML, Meta-SGD^48^, and Meta-Curvature^49^ with the baseline. When using MAML alone, precision is higher than baseline and Dice and Recall score is slightly lower. The method incorporating Meta-Curvature to the baseline has the highest precision evaluation, but the average of all evaluations showed that it is lower than the proposed method using BM-KFO. This suggests that the proposed BM-KFO approach significantly outperforms other methods.

**Table 5:** Result of experimenting with various meta-learning methods without loss function.

Method	Dice score	Precision	Recall	Mean
SSL-ALPNet	73.42	78.43	69.87	73.90
SSL-ALPNet + MAML	69.50	80.79	62.32	70.87
SSL-ALPNet + Meta-SGD^48^	73.00	82.23	70.20	75.14
SSL-ALPNet + Meta-curvature^49^	80.00	**96.30**	71.80	82.70
Proposed method(+ BM-KFO)	**83.40**	87.10	**81.01**	**83.83**

Significant values are in [bold].

### Qualitative evaluation

**Figure 5:**
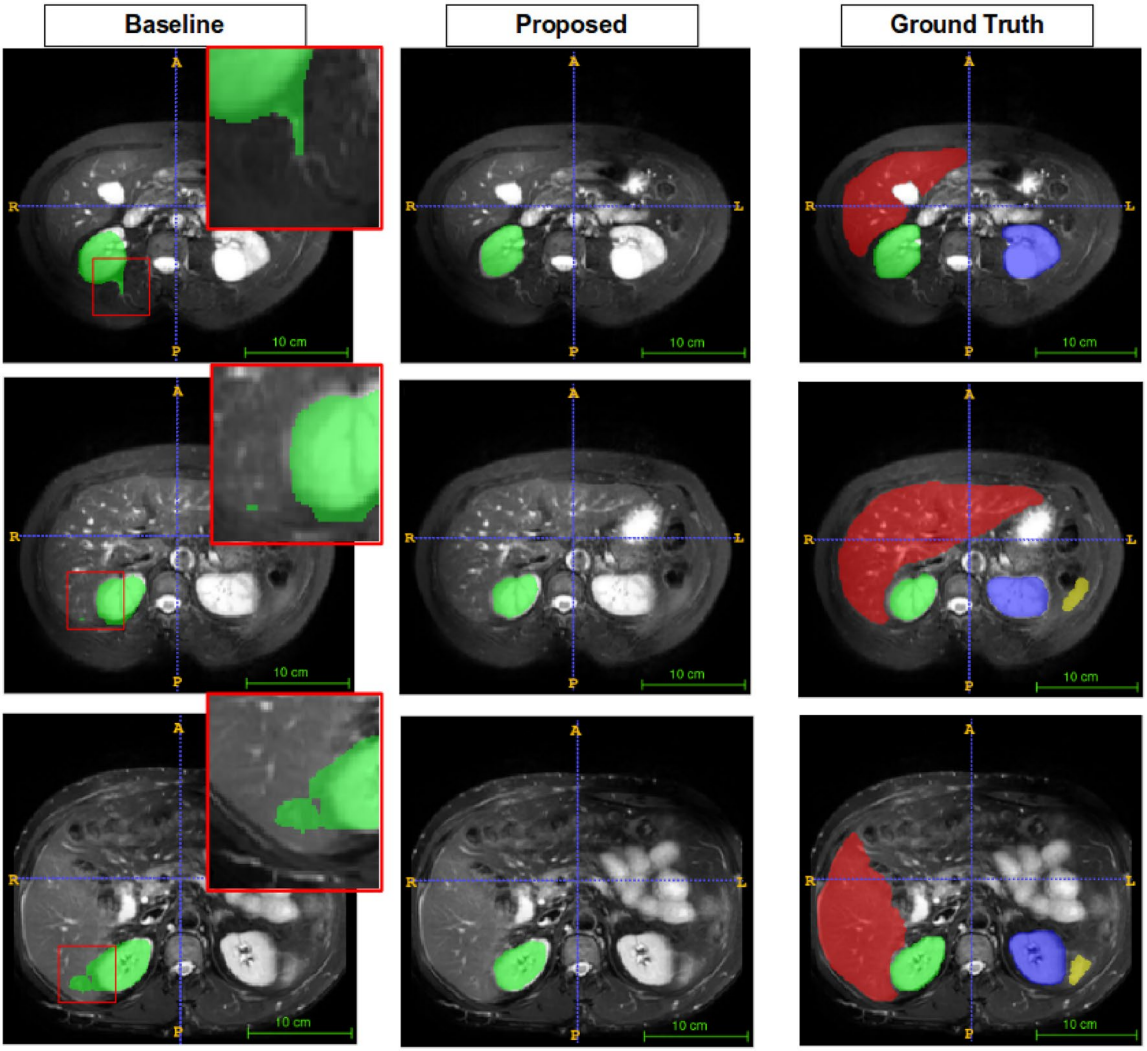
Visual comparison to right kidney.

**Figure 6:**
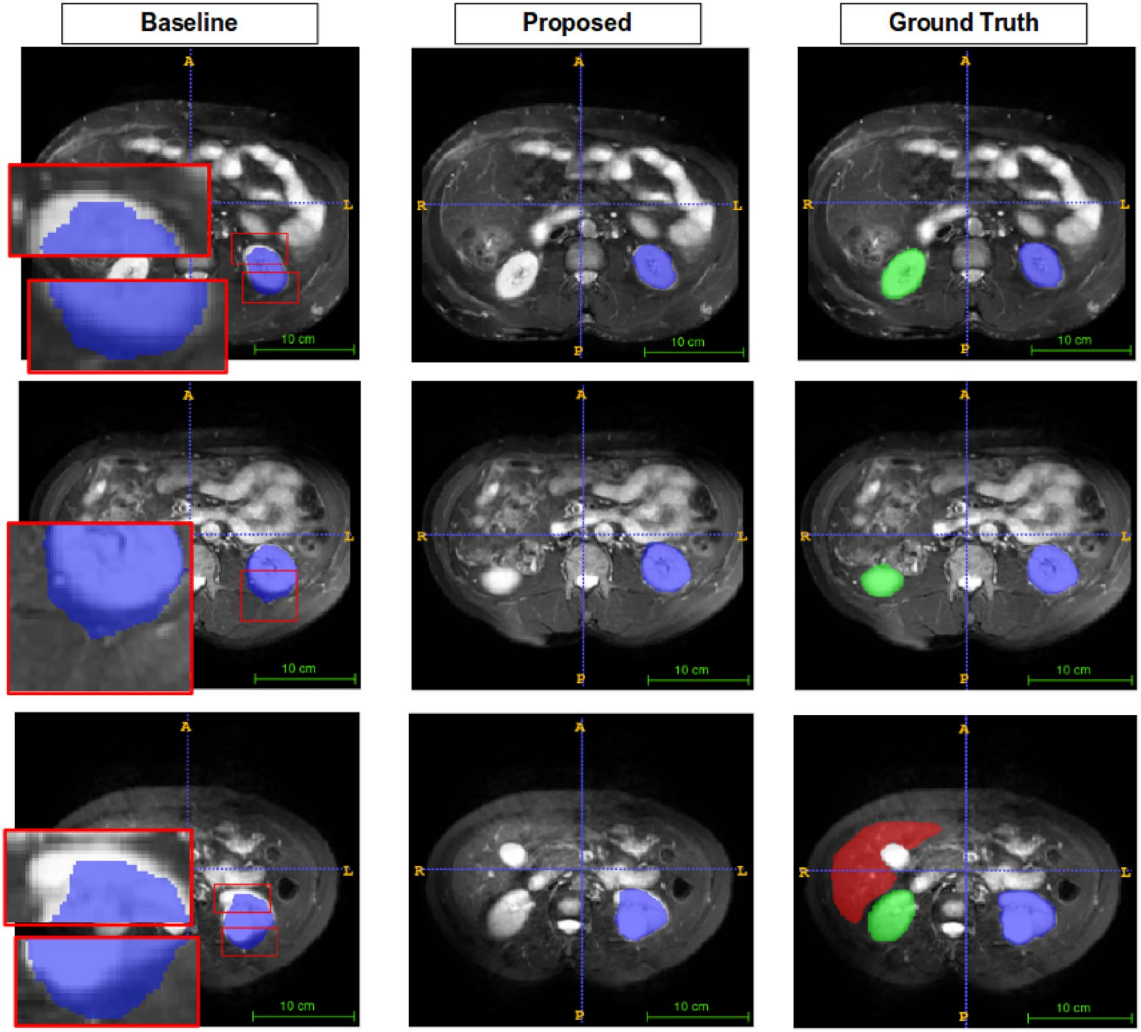
Visual comparison to left kidney. The baseline does not predict segments well, but the proposed method predicts segments well despite of only few data available.

**Figure 7:**
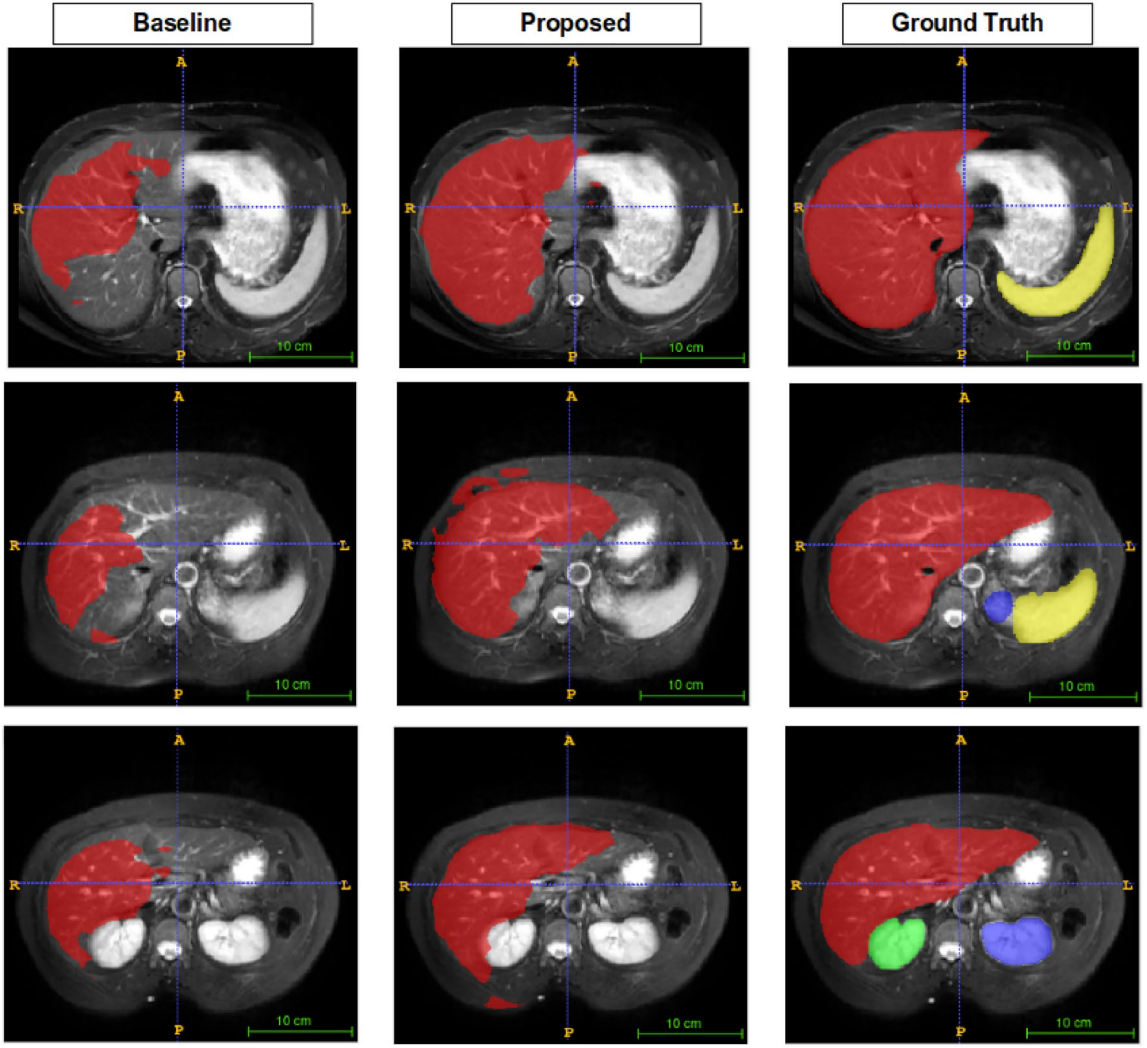
Visual comparison to the liver. It has various shapes and low brightness. The baseline does not predict accurately, but the proposed method predicts shape-aware direction.

**Figure 8:**
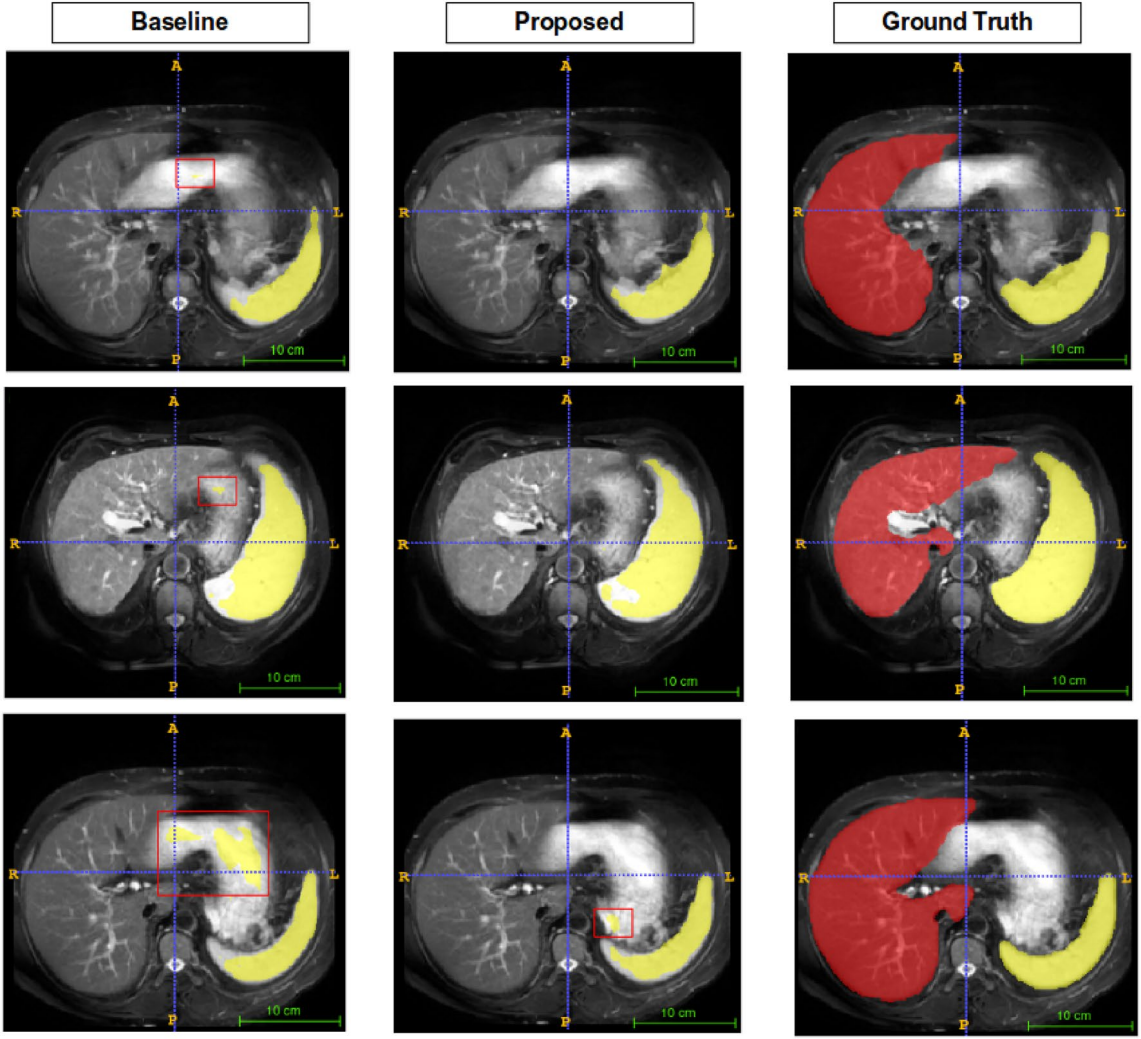
Visual comparison to the spleen. Despite of confused boundary problem, the proposed method predicts shape-aware direction.

Figures 5, 6, 7, and 8 show visual comparison with SSL-ALPNet^31^, proposed method, and corresponding ground truth segmentation on the setting 2. For all experiments, the baseline did not consider the shape of organs. On the other hand, the proposed BM-KFO prediction preserved the shape of the object through fast optimization.

As shown in Fig. 5, the proposed method accurately segmented the right kidney, but the baseline produced an irregular shape. Compared to the baseline methods, the proposed method better focused on the difference in boundaries and the shape of organs, and also produced better results in various shapes and sizes.

As shown in Fig. 6, the shape of the left and right kidneys is closer to GT.

Figure 7 compares the segmentation performance of the liver, which has various shapes per person. For this reason, it is hard to optimize quickly using few-shot learning scheme. Predictions of baseline did not cover corresponding ground truth liver. This class shape variance is still a challenging issue in few-shot learning research. But, we observe that the proposed method segments the liver relatively well.

Figure 8 is a segmented image of the spleen. It has a problem that other organs have similar brightness values. This feature confuses the network and eventually leads to noisy predictions. Baseline suffered from this problem, but the proposed method shows shape-aware outputs.

## Conclusion

In this paper, we presented the BM-KFO framework and AHD-Loss for meta-learning-based medical image segmentation. The proposed method addresses the problem of small data in image segmentation by learning a bidirectional few-shot task that optimizes both the model parameters and the optimizer parameters during training. We applied Kronecker factored and Hausdorff distance loss for efficient segmentation of medical images as well as parameter optimization. By combining these methods with meta-learning, we improved the efficiency of parameter computation and the accuracy of semantic segmentation in few-shot tasks for medical image segmentation. Moreover, this approach may be useful for identifying unseen lesions in medical applications. Compared to existing state-of-the-art methods, the proposed meta-learning framework for medical image segmentation demonstrated an average performance improvement of 1.2%, BM-KFO method by 9.07%, and AHD-Loss function by 2.54%, while producing faster inference and better segmentation results. Although the proposed method increases training time costs due to bidirectional optimization of the model and optimizer for few-shot parameters during training, this can be addressed by changing the structure of the network architecture and improving hardware. Additionally, as the learning time is acceptable in real-world few-shot tasks, the meta-learning approach can assist in the evaluation of medical images across various domains. Thus, the development of meta-medical applications becomes an important field for future research.

## Data Availability

All data generated or analyzed during this study are included in this published article.
